# A Universal Mathematical Methodology in Characterization of Materials for Tailored Design of Porous Surfaces

**DOI:** 10.3389/fchem.2020.601132

**Published:** 2021-01-26

**Authors:** Muhammad Burhan, Faheem Hassan Akhtar, Qian Chen, Muhammad Wakil Shahzad, Doskhan Ybyraiymkul, Kim Choon Ng

**Affiliations:** ^1^Water Desalination and Reuse Centre (WDRC), Biological and Environmental Science & Engineering, King Abdullah University of Science and Technology (KAUST), Thuwal, Saudi Arabia; ^2^Department of Mechanical and Construction Engineering, Northumbria University, Newcastle upon Tyne, United Kingdom

**Keywords:** adsorption, isotherm, energy distribution, adsorbent, physical adsorption, adsorption energy

## Abstract

Understanding adsorption phenomena is essential to optimize and customize the energy transformation in numerous industrial and environmental processes. The complex and heterogeneous structure of the adsorbent surface and the distinct interaction of adsorbent-adsorbate pairs are attributed to the diverse response of adsorption phenomena, measured by the state diagrams of adsorption uptake known as adsorption isotherms. To understand various forms of adsorption isotherms, the surface characteristics of the adsorbent surface with the heterogeneity of adsorption energy sites must be analyzed so that they can be modified for the tailored response of the material. Conventionally, such material synthesis is based on chemical recipes or post-treatment. However, if the adsorbent's surface characteristics and heterogeneity are known, then a directed change in the material structure can be planned for the desired results in the adsorption processes. In this paper, a theoretical and mathematical methodology is discussed to analyze the structure of various adsorbents in terms of the distribution of their adsorption energy sites. The change in their surface is then analyzed, which results in the tailored or customized response of the material.

## Introduction

Adsorption phenomena have numerous industrial and environmental applications, such as desalination, cooling, wastewater treatment, gas or heat storage, and air purification (Burhan et al., 2016a, 2017, 2018a, 2019a, 2020; Xie et al., 2018; Oh et al., 2019; Shahzad et al., 2019a,b; Zhao et al., 2020). Such processes involve distinct interaction of adsorbate molecules over the porous surface of adsorbent material. These adsorbate molecules are accommodated in either single or multilayer formations if favorable adsorbent energy sites are available. The adsorbent-adsorbate pair and their unique interaction according to available adsorption energy sites result in the processes mentioned above and the energy transformation (Antonelli et al., 1992; Zhao et al., 1998). For adsorption cooling, the bulk energy is lost when adsorbate molecules are attracted to adsorption sites, resulting in bulk temperature loss (Uyun et al., 2010; Burhan et al., 2019b; Chen J. L. et al., 2020). Similarly, in adsorption desalination, pure adsorbate molecules are separated from bulk fluid due to their attraction toward the adsorption sites. They are then set free with the energy supply that results in potable water (Shahzad et al., 2017; Chen Q. et al., 2020). The quality of energy supplied to the adsorption system is critical as it is dictated by the unique interaction of the adsorbent-adsorbate pair and the adsorption energy sites that are available to the adsorbate molecules. In adsorption dehumidification, the quality and amount of energy required for regeneration depends upon adsorption energy sites that capture adsorbate molecules from the mainstream (Yang K. S. et al., 2017; Yang W. et al., 2017; Burhan et al., 2018b).

**Figure 1:**
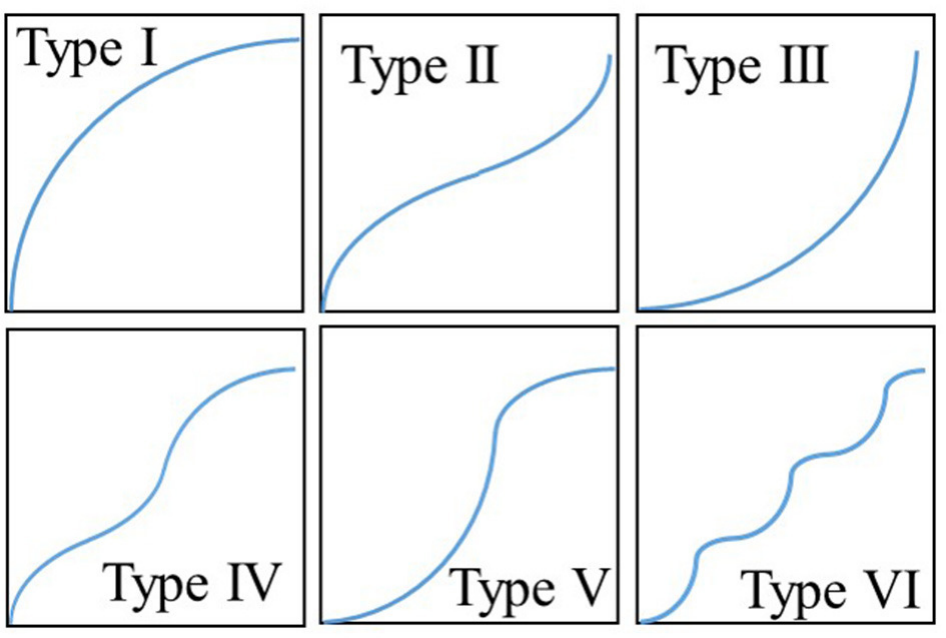
IUPAC Six types of isotherms.

Every adsorbent has diverse surface characteristics defined by the pore size and corresponding adsorption energy sites. Depending on these adsorption energy sites' heterogeneity, every single adsorbent-adsorbate has unique behavior characterized by the adsorption isotherms. As per the International Union of Pure and Applied Chemistry (IUPAC) (Schneider, 1995), all of the adsorption isotherms, known till now, are categorized into six types depending upon their shapes, as shown in Figure 1.

Many studies tried to describe the unique behavior of adsorbent-adsorbate pair interaction. However, due to the availability and heterogeneity of adsorption energy sites for each adsorbate interaction, researchers could not generalize adsorption isotherms. The classical models of Langmuir, Toth, and Henry (Tóth, 1994; von Gemmingen, 2005; Foo and Hameed, 2010) can define the adsorption characteristics when the adsorbate concentration is low near the adsorption sites. On the other hand, Dubinin-Astakhov (DA) and Dubinin-Radushkevich (DR) models were able to depict the behavior of the adsorbent surface when the concentration of adsorbate molecules was near-saturated in value (Dubinin and Astakhov, 1971a,b,c; Burhan et al., 2016b). The previous studies were focused on the characterization of adsorption based on the type of isotherm. Some researchers defined separate mathematical models for each isotherm type (Yahia et al., 2013). However, many of the studies focused on the generalization of similar types of isotherms (Khalfaoui et al., 2003; Chakraborty and Sun, 2014). But none of the models was able to generalize the adsorption theory and define all of the six isotherms types until we introduced the universal isotherm model (Ng et al., 2017; Burhan et al., 2018c).

The main emphasis of the generalization of adsorption isotherms was to understand adsorption phenomena so that they can be tailored for required needs. The porous adsorbent's surface characteristics can be modified through several processes such as acidification, heat, or chemical treatment (Baker and Sing, 1976; Naono et al., 1980; Ishikawa et al., 1996; Shioji et al., 2001; Toor and Jin, 2012; Burhan et al., 2016c,d; Jiang et al., 2018; Liu et al., 2020). The pore structure and size are also associated with the adsorption characteristics (Burhan et al., 2019c), and response can be modified either by post-treatment of material or with the new recipe of the material. The main idea of material synthesis is to improve and customize the material's performance for a certain application. Therefore, it is very important to understand the structure and characteristics of the adsorbent surface so that the required modifications can be made for the tailored performance of the material. In this paper, a theoretical and mathematical methodology is discussed and explained to analyze the adsorption phenomena with their available adsorption energy sites. With the distribution curve of energy sites over the adsorbent's heterogeneous porous surface, the adsorption phenomena have been analyzed by understanding the response of the adsorbent in terms of availability of its energy sites during interaction with the adsorbate molecules. Moreover, a change in the response of the adsorbent is also explained in terms of adjustment in the distribution curve of adsorption energy sites or adsorbent structure, which resulted in such a tailored material response.

## Methods and Materials

The main principle for the adsorption phenomena to occur depends on the availability of the adsorption energy sites, which depends on the critical energy ε_*c*_ of the adsorbate molecules, i.e.,

(1) $$\begin{array}{l} \varepsilon_{c}=-RT\ln Kp \end{array}$$

Where *T* and *p* represent the temperature and the pressure of adsorbate molecules approaching adsorption energy sites. However, *K* represents the ratio of the rate of adsorption and equilibrium constants, i.e., $K{=}^{{\;K}_{a}}{/}_{K_{d}}$, or in simple form the reciprocal of saturated pressure of adsorbate molecules corresponding to their temperature, i.e., $K{=}^{\;1}{/}_{p_{s}}$. Suppose the critical energy of the adsorbate molecules becomes lower than the energy level of any adsorption site. In that case, these sites become available for adsorption, and the fraction of those available sites determines the amount and rate of adsorbed molecules. Therefore, considering the heterogeneity of the adsorbent surface and the proportionality of the amount of adsorption uptake with its fractional availability of energy sites, the sum of adsorption uptake for all of the available energy sites can give the total adsorption uptake, i.e.,

(2) $$\begin{array}{l} \theta_{t}=\int_{0}^{\infty }\left\{\theta (\varepsilon )X(\varepsilon )\right\}d\varepsilon \end{array}$$

Where *X(*ε*)* represents the energy distribution function (EDF), or fractional availability of particular adsorption energy sites, and θ*(*ε*)* represents the adsorption uptake of molecules by the corresponding adsorption site with energy ε. It is essential to mention here that not all of the adsorption sites are available for adsorbate molecules except ones with an energy level higher than the critical energy ε_*c*_. Therefore, the above expression can be written as:

(3) $$\begin{array}{l} \theta_{t}=\int_{\varepsilon_{c}}^{\infty }\left\{\theta (\varepsilon )X(\varepsilon )\right\}d\varepsilon \end{array}$$

By taking the limit of the adsorption site energy, the localized uptake parameter takes the form of a binary value as:

(4) $$\begin{array}{l} \underset{T\to 0}{\lim}\;\theta (\varepsilon )=\theta_{c}(\varepsilon )=|\begin{array}{c} 0\;for\;\Delta \varepsilon <\varepsilon_{c}\\ 1\;for\;\Delta \varepsilon \geq \varepsilon_{c} \end{array} \end{array}$$

Where Δε represents the difference between adsorption and desorption energy. In simple form, Δε = ε − *h*_*fg*_, i.e., the difference in the adsorption site's energy value and the vaporization energy of the adsorbate molecule. Thus, the adsorption uptake expression can be simplified as

(5) $$\begin{array}{l} \theta_{t}=\int_{\varepsilon_{c}}^{\infty }X(\varepsilon )d\varepsilon \end{array}$$

The above expression states that the total adsorption uptake mainly relies on the distribution of the adsorption energy sites. Therefore, if the adsorption uptake is known, the porous structure and the adsorbent's surface heterogeneity can be visualized and analyzed mathematically. On the other hand, as highlighted before, the real need to understand the porous adsorbent structure is to design the material for tailored performance needs.

Hence, to truly capture the adsorption phenomena, it is important to accurately predict the distribution and heterogeneity of energy sites of the adsorbent surface. The symmetrical Gaussian function is considered to be representing the distribution of energy sites over the porous adsorbent surface.

(6) $$\begin{array}{l} X{\left(\varepsilon \right)}_{i}={\left\{\frac{\exp\left(\frac{\Delta \varepsilon -\varepsilon_{oi}}{m_{i}}\right)}{m_{i}{\left[1+\exp\left(\frac{\Delta \varepsilon -\varepsilon_{oi}}{m_{i}}\right)\right]}^{2}}\right\}}_{i} \end{array}$$

The terms ε_o_ and *m* represent the median energy level and the surface heterogeneity of the adsorbent structure. However, the complex porous adsorbent structure cannot be represented with a single distribution. Therefore, a probability function α is coupled with the classic Gaussian function to represent the probability of a group of adsorption energy sites instead of a single group of energy sites.

(7) $$\begin{array}{l} X(\varepsilon )=\sum_{i=1}^{n}\alpha_{i}\;{\left\{\frac{\exp\left(\frac{\Delta \varepsilon -\varepsilon_{oi}}{m_{i}}\right)}{m_{i}{\left[1+\exp\left(\frac{\Delta \varepsilon -\varepsilon_{oi}}{m_{i}}\right)\right]}^{2}}\right\}}_{i} \end{array}$$

With the introduction of the above Gaussian function of Equation (7) in Equation (5), the universal isotherm model can be written as (Ng et al., 2017):

$$\begin{array}{l} \theta_{t}=\alpha_{1}\;\left[\frac{{\left(\frac{p}{p_{s}}\exp\left(\frac{\varepsilon_{o1}}{RT}\right)\right)}^{\frac{RT}{m1}}}{1+{\left(\frac{p}{p_{s}}\exp\left(\frac{\varepsilon_{o1}}{RT}\right)\right)}^{\frac{RT}{m1}}}\right]\\ \;+\alpha_{2}\;\left[\frac{{\left(\frac{p}{p_{s}}\exp\left(\frac{\varepsilon_{o2}}{RT}\right)\right)}^{\frac{RT}{m2}}}{1+{\left(\frac{p}{p_{s}}\exp\left(\frac{\varepsilon_{o2}}{RT}\right)\right)}^{\frac{RT}{m2}}}\right]\\ \;+\alpha_{3}\;\left[\frac{{\left(\frac{p}{p_{s}}\exp\left(\frac{\varepsilon_{o3}}{RT}\right)\right)}^{\frac{RT}{m3}}}{1+{\left(\frac{p}{p_{s}}\exp\left(\frac{\varepsilon_{o3}}{RT}\right)\right)}^{\frac{RT}{m3}}}\right]\;......\;\\ \;\alpha_{n}\;\left[\frac{{\left(\frac{p}{p_{s}}\exp\left(\frac{\varepsilon_{on}}{RT}\right)\right)}^{\frac{RT}{mn}}}{1+{\left(\frac{p}{p_{s}}\exp\left(\frac{\varepsilon_{on}}{RT}\right)\right)}^{\frac{RT}{mn}}}\right] \end{array}$$

**Figure 2:**
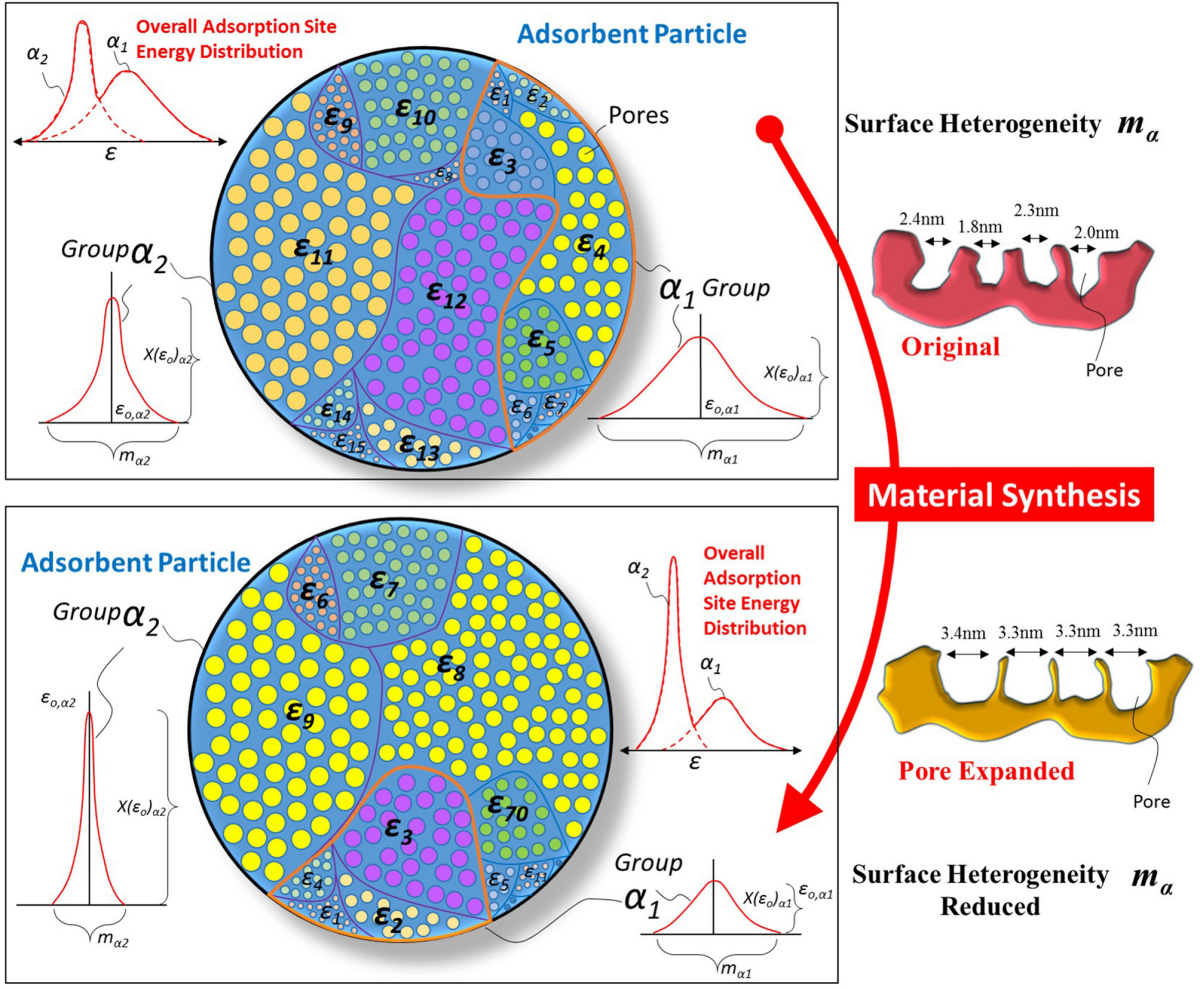
Schematic of the porous adsorbent surface with the presence of adsorption energy sites distribution and possible change in it after material synthesis. The material synthesis caused the expansion of pores that resulted in a difference in the material's topography. The surface heterogeneity of synthesized material is reduced as fractional availability *X(*ε*)* of larger pores is higher when smaller pores were expanded and as a result, the overall spread “*m*” of energy distribution is reduced.

The main application of the above isotherm equation is to trace the adsorption uptake. If the adsorption uptake is known, then the characteristic parameters of the adsorbent surface can also be determined, leading to understanding the distribution of adsorption energy sites over the porous surface. Figure 2 shows the schematic of a porous adsorbent surface with adsorption energy site distribution. The adsorption sites are available with different adsorption energy levels ε, and the distribution of these energy sites as a function of their availability is given by Equation (7). The presence of many adsorption energy sites results in higher surface heterogeneity “*m*” of the porous surface. And the high surface heterogeneity enables the material to respond to a broad range of adsorbate molecules' concentration/pressure ratio.

Most of the time, the adsorbent's response is required at a certain concentration/pressure ratio of adsorbate molecules, which requires a change in the structure and topography of the porous adsorbent surface. Many material synthesis techniques are available that can cause the pores' expansion of the porous surface resulting in a change of the adsorption sites energy distribution. If the desired change in the adsorption sites' energy distribution is known, then the material can be tailored to achieve the required application. The proposed theoretical framework enables scientists to understand the required energy distribution to achieve particular isotherm needs. Figure 2 shows how material synthesis techniques expand the adsorption surface's pores and reduce surface heterogeneity. Such reduced heterogeneity causes high availability of specific adsorption energy sites resulting in increased adsorbent uptake at a certain concentration/pressure ratio of adsorbate molecules. A detailed discussion of the adsorbent energy distribution and its effect on adsorption uptake is presented in the next section.

## Results and Discussion

**Figure 3:**
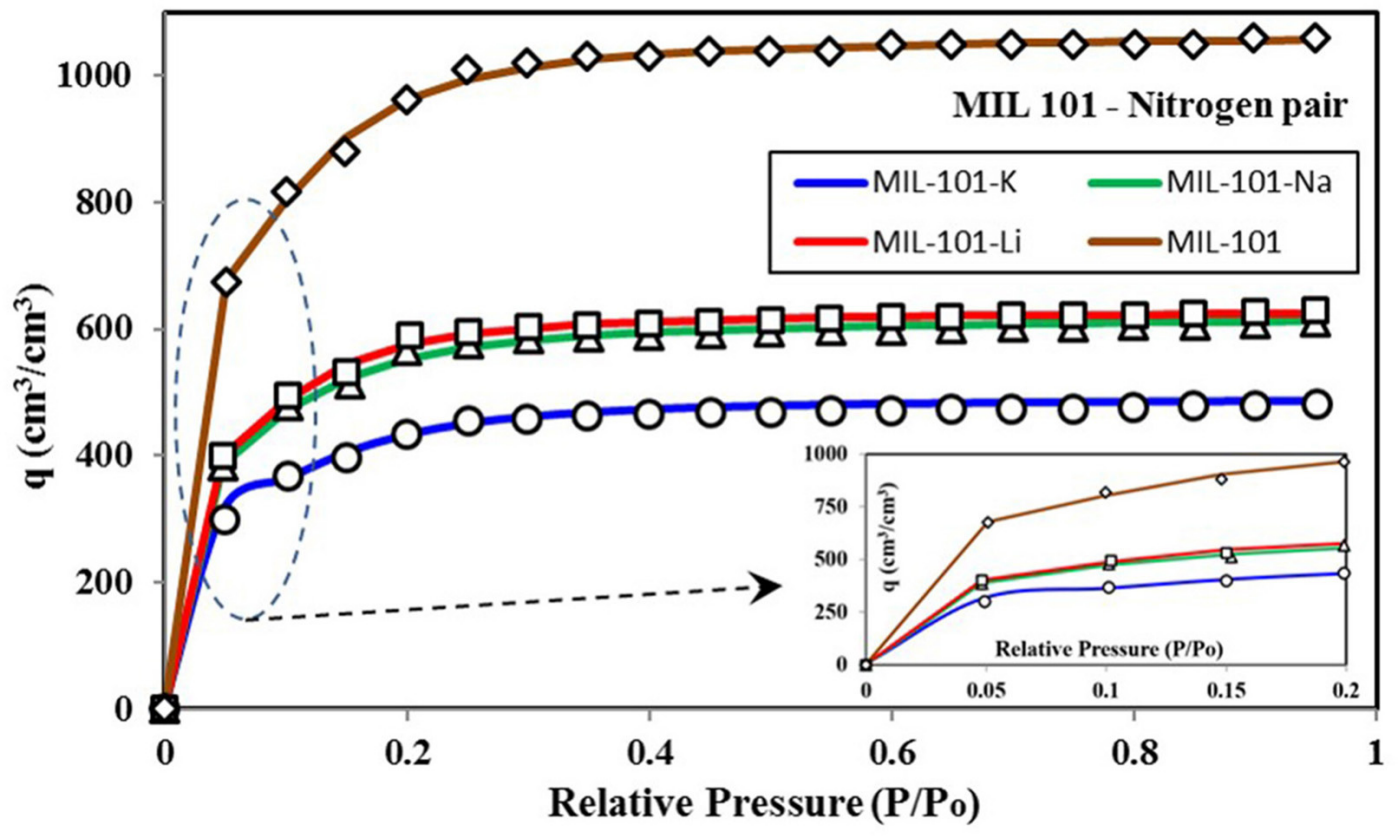
Isotherms for MIL-101 variants paired with nitrogen as the adsorbate. The expanded section shows a small saturation of uptake at lower pressure ratios.

To look into the energy distribution, it is crucial to understand the universal isotherm model's insight. In this manuscript, all of the isotherms considered are based upon nitrogen as adsorbate for a fair comparison. Because nitrogen-based isotherms are frequently discussed in the literature, a large data set is available, which can provide a detailed discussion on the proposed methodology's potential. However, the present methodology and model apply to any adsorbent-adsorbate pair, not only nitrogen. Figure 3 shows the nitrogen isotherms of different variants of MIL-101 adsorbent (Teo et al., 2017), and the corresponding key values of parameters of the universal isotherm model used to fit all of the four isotherms are given in Appendix Table 1. All of the four data show TYPE-V1 behavior as there is a small saturation step seen at a relative pressure of 0.1. Besides, an early saturation of all of the four isotherms can be seen at a relative pressure ratio of 0.3 only. Due to Type-IV isotherms' dual step behavior, as shown in Figure 1, two terms of the universal isotherm models were used to fit the data, which also show two groups of adsorption energy sites responsible for the adsorption uptake. The term with a higher median energy level is responsible for the low-pressure adsorption uptake. These energy sites become easily available when critical energy ε_c_ goes low with an increase in concentration. Also, this group of low-pressure adsorption sites is responsible for 60–65% (α_1_) of the total energy sites and the adsorption uptake.

**Figure 4:**
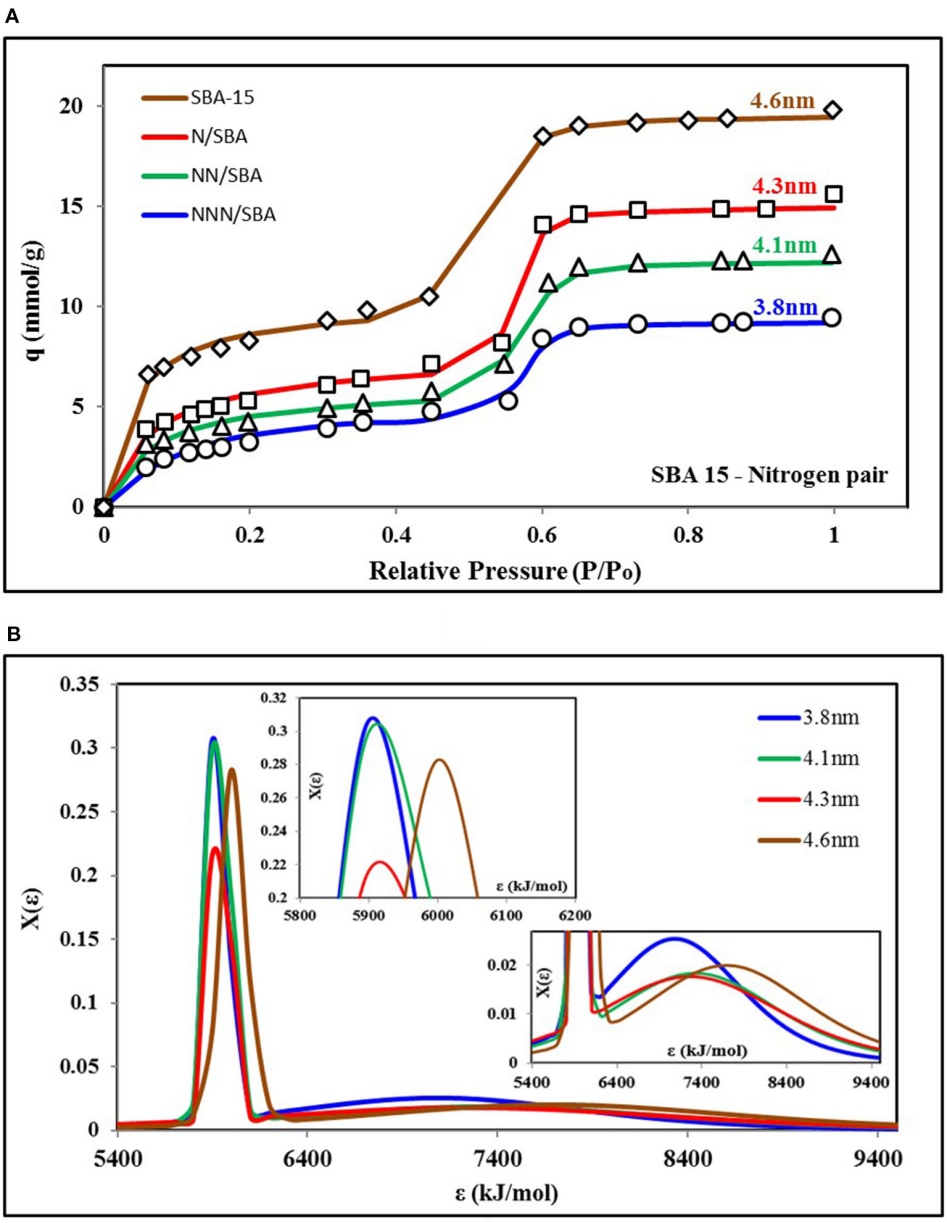
**(A)** Isotherms and **(B)** adsorption sites energy distribution for SBA-15 variants paired with nitrogen as the adsorbate. The material is synthesized for pore expansion that resulted in a change in energy distribution for corresponding isotherms. Here, pore expansion caused an increase in smaller pores' availability and expanded distribution of energy sites. However, surface heterogeneity doesn't have any significant change.

Figure 4A shows the nitrogen isotherms of different variants of SBA-15 adsorbent (Abdouss et al., 2014) and the corresponding key values of parameters of universal isotherm model used to fit all of the four isotherms are given in Appendix Table 2. Similar to the isotherms shown in Figure 3, two terms of the universal isotherm models were used to fit the data, depicting the two groups of adsorption energy sites responsible for the adsorption uptake. Also, all of the four curves also show TYPE-V1 behavior as there is a significant saturation step seen with the relative pressure of 0.5. On the other hand, both adsorption site groups are responsible for almost 50% of the share of surface occupancy and the adsorption uptake. Nonetheless, all of the four isotherms showed similar behavior in response to relative pressure but with different highest levels of total uptake. Such high uptake is due to pore expansion of the material with increased pore volume. However, the pore expansion process did not cause any significant change in the adsorbent surface's heterogeneity and structure. This can be seen in Figure 4B as three isotherms have almost the same median energy level ε_o_ and heterogeneity spread “*m*.”

**Figure 5:**
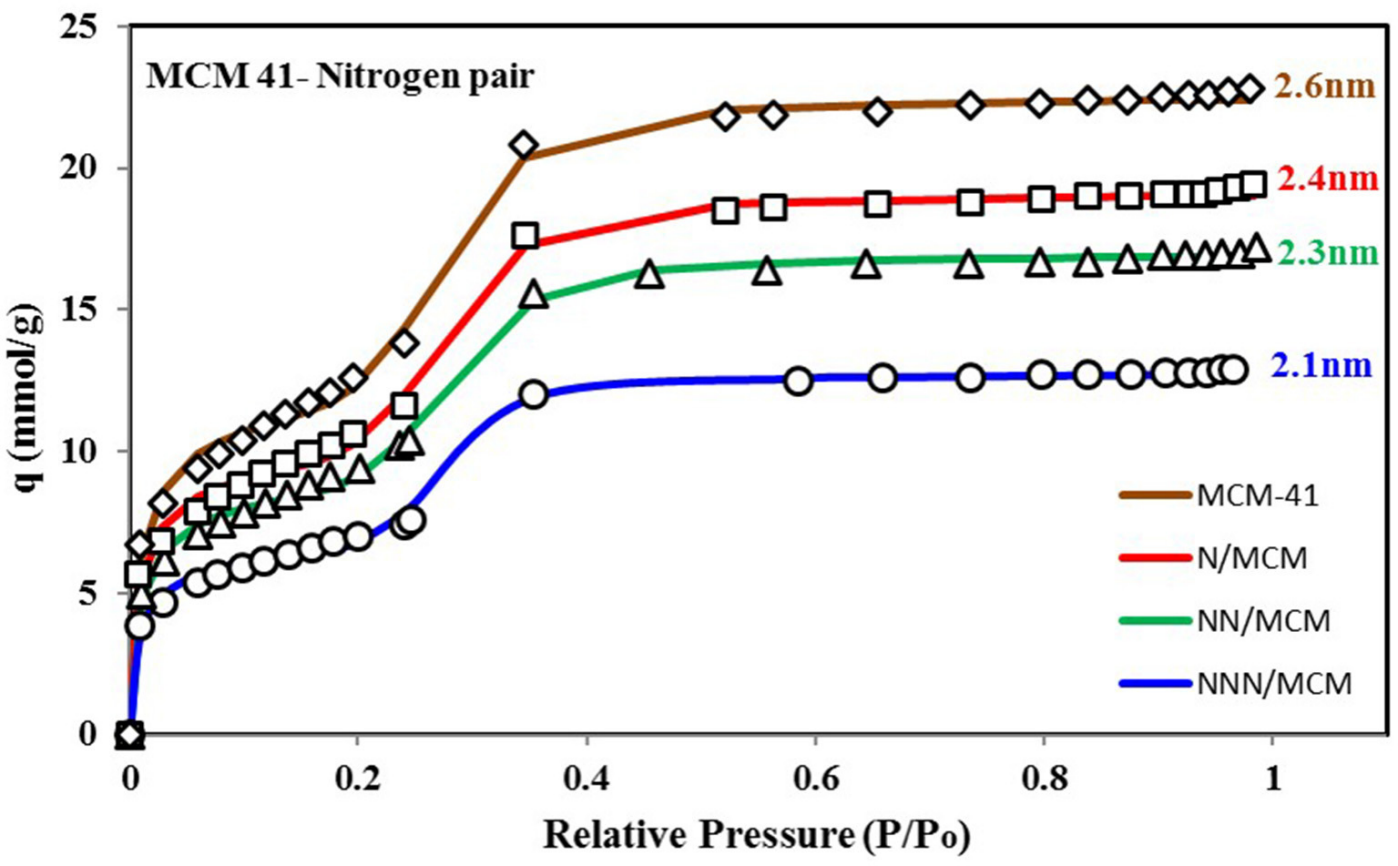
Isotherms for MCM-41 variants with pore expansion when paired with nitrogen as adsorbate. The pore expansion caused an increase in uptake with the same response against relative pressure, which can be verified from the energy distribution plot with the same median energy level of all four variants.

**Figure 6:**
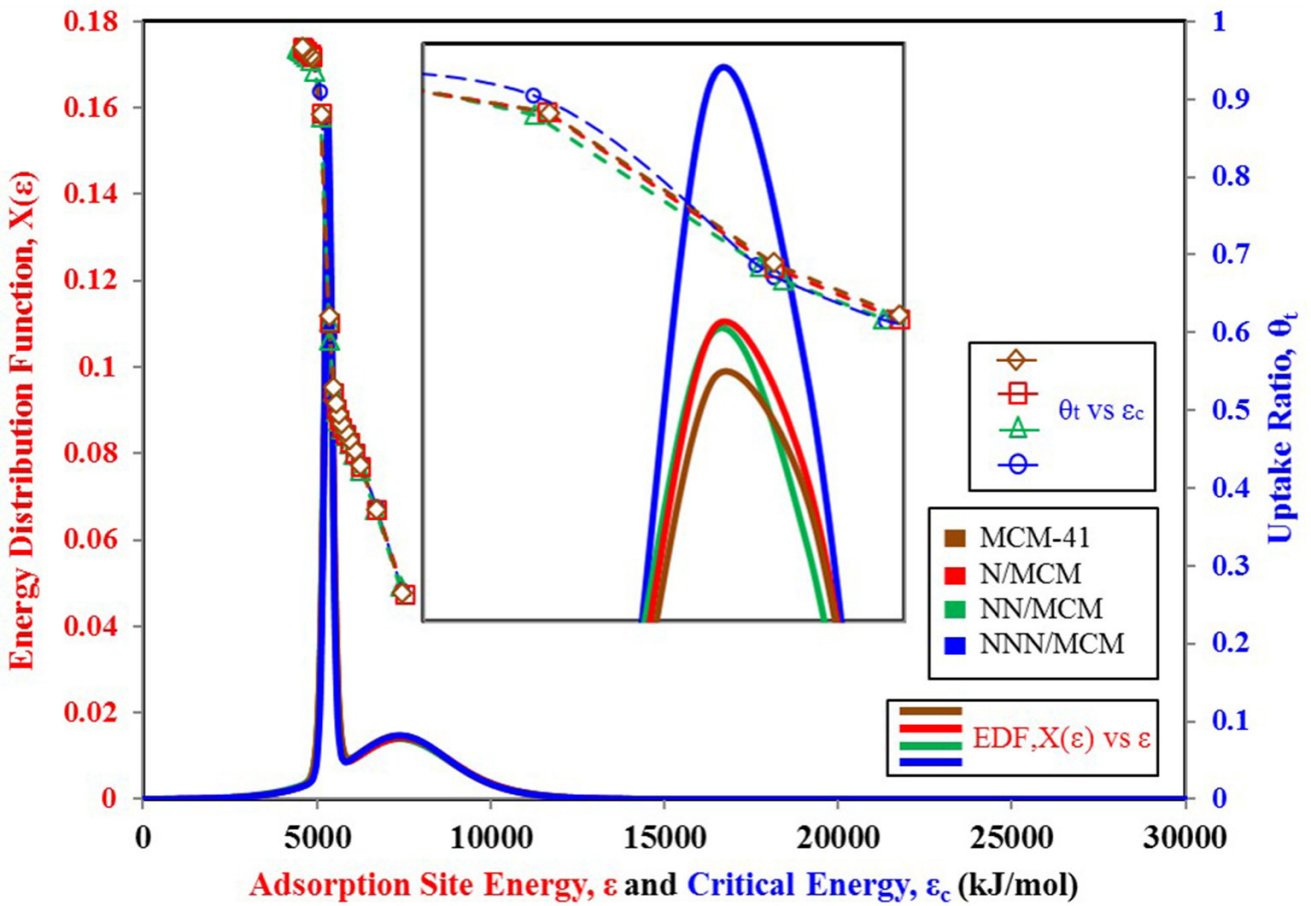
Adsorption sites energy distribution for MCM-41 variants paired with nitrogen as adsorbate.

It is vital to analyze the graphical form of energy distribution function EDF associated with the universal isotherm model's key parameters. Figure 5 shows the nitrogen isotherms of other variants of MCM-41 adsorbent (Abdouss et al., 2014), which is similar in behavior to isotherms shown in Figure 4A as they are also TYPE VI isotherms with dual step saturation. Also, all of the four isotherms are showing a similar response against relative pressure. The energy distribution curves of all four MCM-41 variants are shown in Figure 6. This graphical representation of the adsorption sites' energy distribution has more physical meaning, and it gives a better understanding of the porous surface's surface heterogeneity for analysis adsorption phenomena. As represented by Figure 6, all of the four variants of MCM-41 have the same distribution of adsorption energy sites. All of the four site energy distribution curves are overlapping. Therefore, for better analysis, magnified curves are also shown in the same figure. MCM-41 shows a lesser probability of the median energy level as compared with NNN/MCM despite the fact that the total uptake of MCM-41 is higher than the NNN/MCM. It must be noted here that the higher uptake is linked with the pore size and corresponding higher pore volume, which has the capacity to accommodate a larger number of adsorbate molecules, as with the case of MCM-41. On the other hand, the higher probability of the site energy distribution curve, as is the case of NNN/MCM, is linked with the steeper response of material with changing relative humidity.

**Figure 7:**
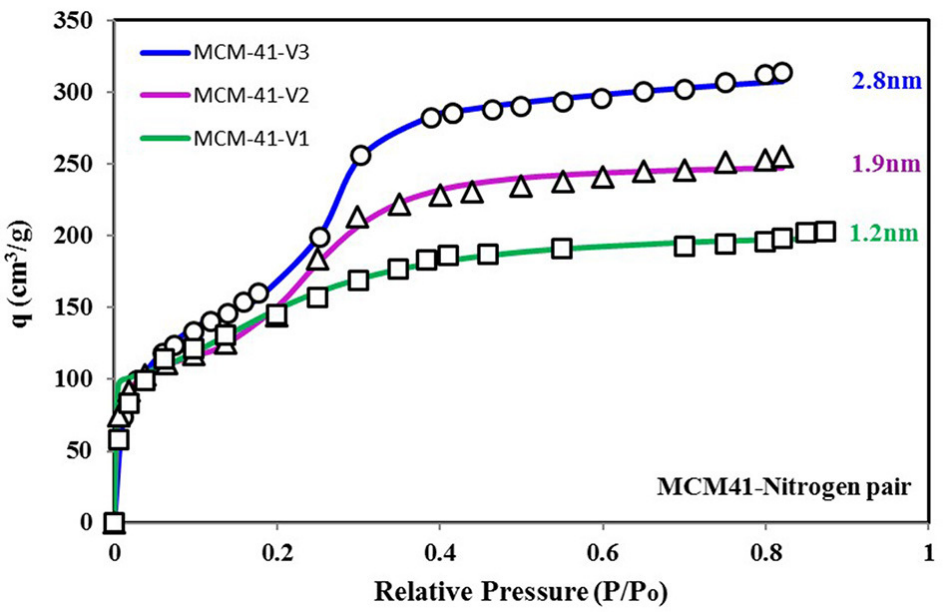
Isotherms for MCM-41 V1, V2, and V3 variants with pore expansion when paired with nitrogen as the adsorbate. The pore expansion caused an increase in uptake. However, the response of the material against relative pressure shifted from smooth to sharp uptake. This transition can be explained from the energy distribution plot, where availability of median energy level is increasing with pore expansion, causing a sharp uptake when the ε_c_ value goes lower than the median energy level.

**Figure 8:**
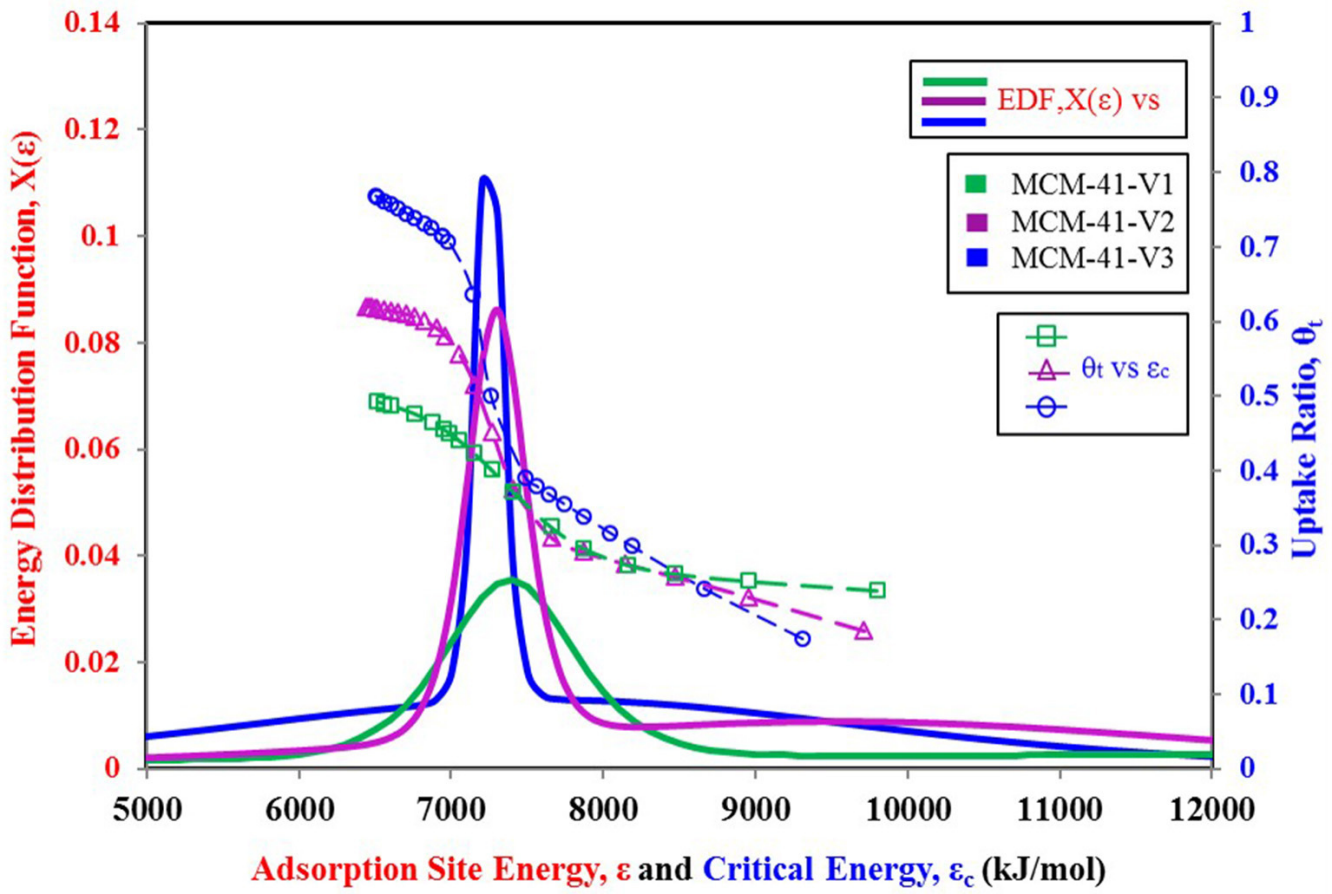
Adsorption sites energy distribution for MCM-41 V1, V2, and V3 variants paired with nitrogen as adsorbate.

To better explain the relationship of the higher probability of site energy distribution curve with the sharp response of material against changing relative pressure, another set of data is shown in Figures 7, 8. Figure 7 shows three more variants of MCM-41 adsorbent (Ng et al., 2013) with a TYPE-VI isotherm. This adsorbent, with larger pore size, has a higher uptake, and apparently, all three isotherm curves respond similarly to each other against the changing relative pressure. However, from Figure 8, it can be seen that the MCM-41-V3 has the highest probability of being at the median energy level as it shows a sharp response toward the changing relative pressure from 0.2 to 0.3. On the other hand, MCM-41-V1 has a lower probability of reaching the median energy level, but with a larger spread of the curve. As a result, a gradual adsorption uptake is observed with changing relative pressure from 0.1 to 0.3. The higher probability of any energy level depicts the availability of such an adsorption site with a large number over the surface. And when the critical energy level ε_c_ of adsorbate molecules goes lower than the median energy level, a large group of adsorption sites becomes available for the adsorption. As a result, a sharp rise in the adsorption uptake is observed and can be observed through θ_*t*_ vs. ε_c_ curves. That is why MCM-41-V3 has a sharp adsorption uptake compared with MCM-41-V1, which has higher surface heterogeneity of adsorption energy sites. However, all of the four curves' median energy levels are almost the same, which means that all three variants respond at almost the same relative pressure value. To modify the response of material for different relative pressure value, the median energy level needs to be changed through post-treatment of the material.

**Figure 9:**
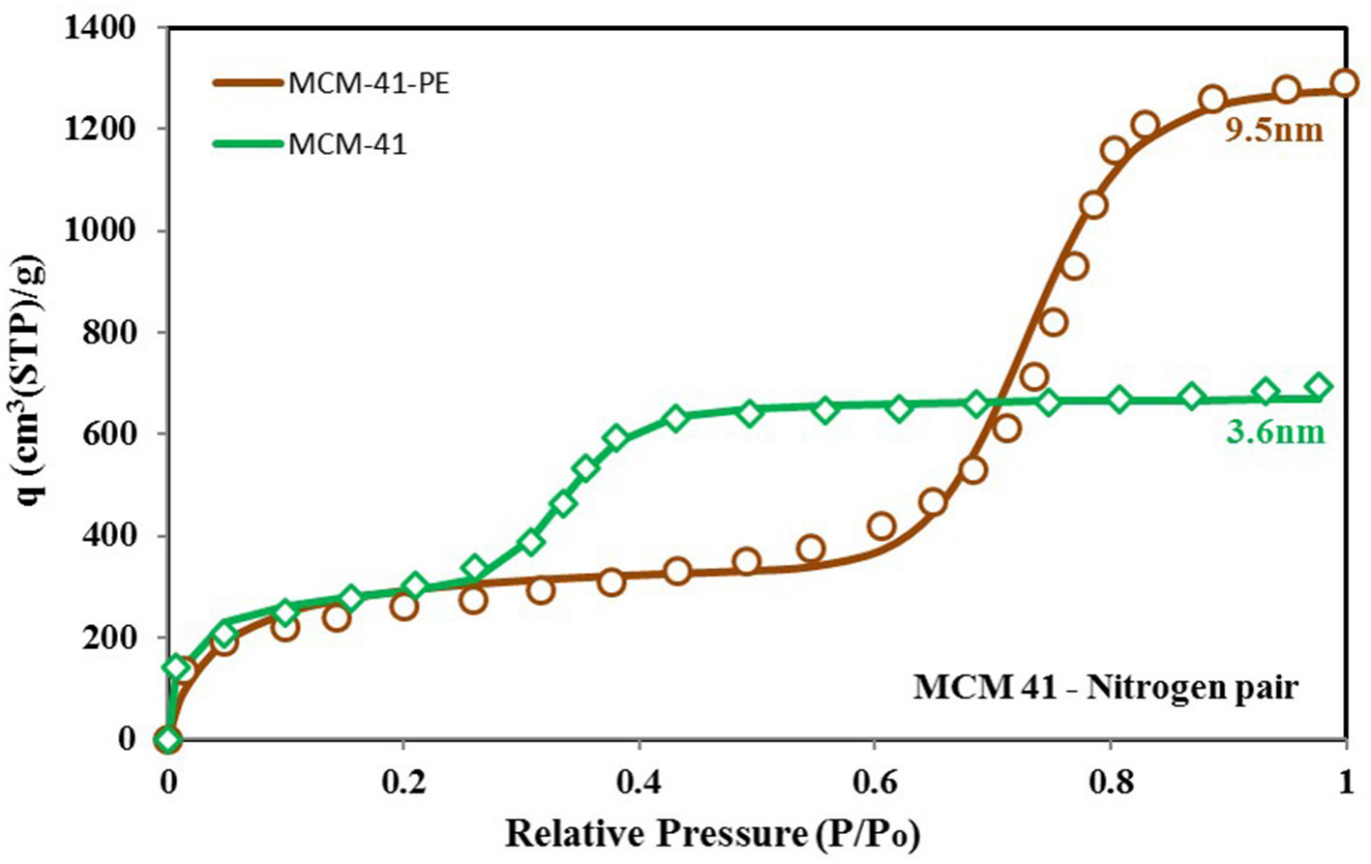
Isotherms for MCM-41 and MCM-41-PE paired with nitrogen as the adsorbate. Unlike the previously shown isotherm cases, pore expansion caused a higher uptake and caused a shift in the material's response against relative pressure. This shift can be linked to the change in the median energy level, presented by the energy distribution function.

**Figure 10:**
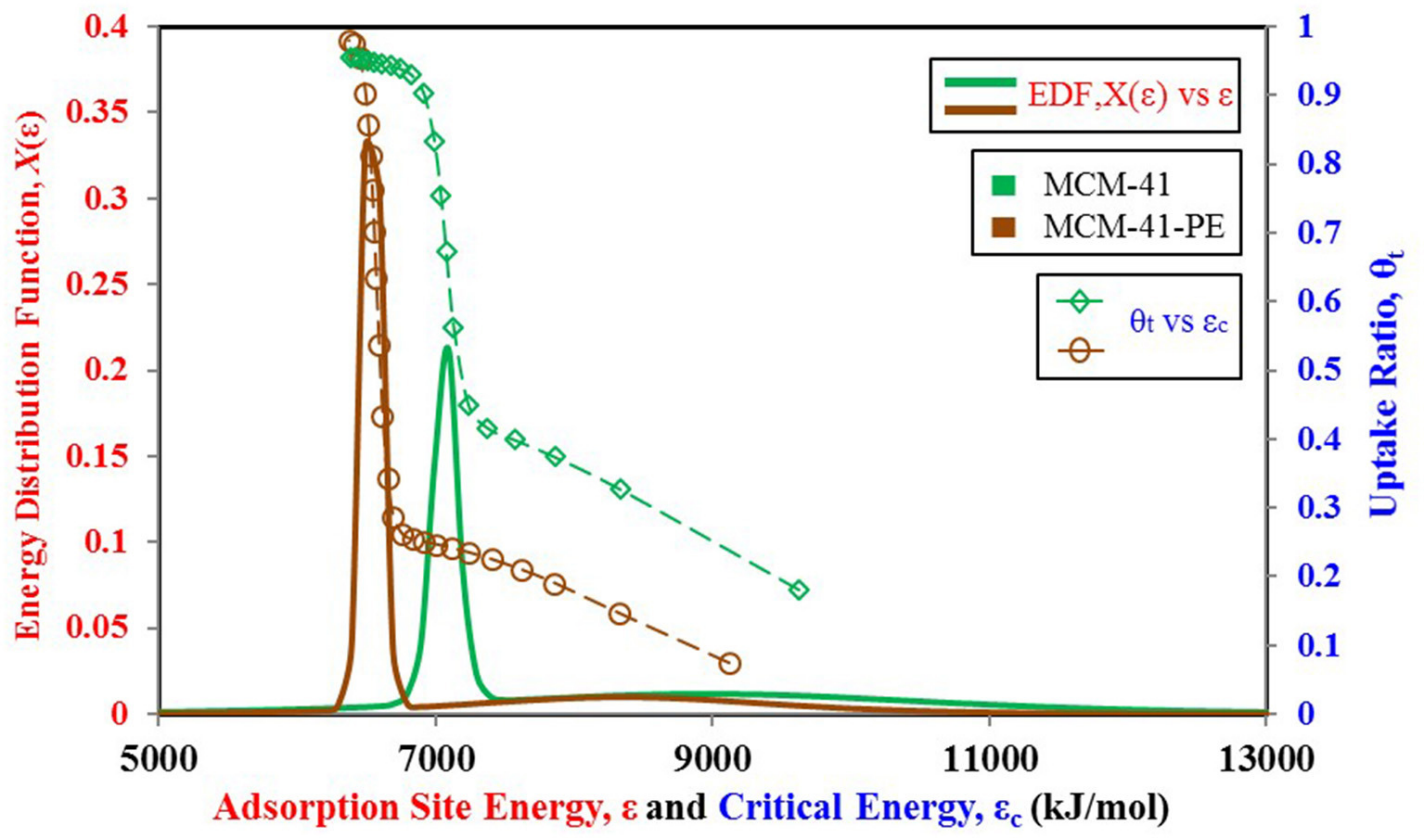
Adsorption sites energy distribution for MCM-41 and MCM-41-PE paired with nitrogen as adsorbate.

From the above discussion, we can conclude that the adsorbent's behavior depends on its surface heterogeneity and the distribution of adsorption energy sites. Therefore, for a tailored response of the material, its surface characteristics can be changed to achieve the desired response and the uptake in the form of adsorption site energy distribution. Figure 9 shows how the response of the MCM-41 material is modified for higher uptake and high relative pressure response by pore expansion (Harlick and Sayari, 2007). The site energy distribution curves of MCM-41 and pore expanded MCM-41-PE are shown in Figure 10. It can be seen that the response of both materials is the same for low relative pressure values, so their site energy distribution curves are also similar to each other. However, to shift the response of the MCM-41, the median energy level is shifted toward a lower energy level, causing the material to shift its response from 0.3 relative pressure value to 0.6. Thus, a tailored response from the adsorption material can be achieved by chemists through the selective design of the materials from different surface treatment techniques such as heat treatment (Naono et al., 1980; Shioji et al., 2001), chemical treatment (Ishikawa et al., 1996), acidification (Toor and Jin, 2012), and the analysis of corresponding changes in the distribution of adsorption energy sites.

## Conclusion

By understanding the distribution of adsorption energy sites and surface heterogeneity of adsorption, the diverse behavior of adsorption phenomena can be captured clearly. With the relationship between available adsorption energy sites and the critical energy of adsorbate molecules, along with surface heterogeneity, the adsorption process can be customized for the tailored need of uptake and response during various chemical or physical processes. Unlike the conventional approach of material synthesis, the structure of the material in terms of the distribution of adsorption energy sites can be understood first, and the adjustment needed in the distribution of adsorption sites to get the customized response of the material must be kept in focus for targeted material synthesis. With such an understanding of adsorbent heterogeneous surfaces, the new adsorbent-adsorbate pairs can be configured with the desired isotherms for boundless applications.

## Data Availability Statement

The original contributions presented in the study are included in the article/Supplementary Materials, further inquiries can be directed to the corresponding author/s.

## Author Contributions

MB provided theory and mathematical model and wrote manuscript. MB, FA, QC, MS, and DY collected data. MB and KN analyzed data. All authors contributed to the article and approved the submitted version.

## Conflict of Interest

The authors declare that the research was conducted in the absence of any commercial or financial relationships that could be construed as a potential conflict of interest.
