# Supplementary material for: A Universal Mathematical Methodology in Characterization of Materials for Tailored Design of Porous Surfaces
Source: Front Chem. 2021 Jan 26;8:601132. doi: 10.3389/fchem.2020.601132 (PMC7870507; doi:10.3389/fchem.2020.601132)
Supplement: Supplementary file 1 [file Table_1.DOCX]

# Appendix

Table 1: Key values of parameters of MIL 101 - Nitrogen isotherm

| **Material Type** | **Parameter** | **Value** | **Parameter** | **Value** |
| --- | --- | --- | --- | --- |
| MIL-101-K  q*(cm^3^/cm^3^) = 500 | ε_o1_ (J/mol) | 9137.4 | ε_o2_ (J/mol) | 1282.2 |
|  | m1 (J/mol) | 2816.0 | m2 (J/mol) | 269.8 |
|  | α_1_ | 0.6527 | α_2_ | 0.3473 |
|  | BET (m^2^/g) | 1547.33 | Pore Vol. (cm^3^/g) | 0.72 |
| MIL-101-Na  q*(cm^3^/cm^3^) = 640 | ε_o1_ (J/mol) | 6837.4 | ε_o2_ (J/mol) | 1482.2 |
|  | m1 (J/mol) | 2516.0 | m2 (J/mol) | 329.8 |
|  | α_1_ | 0.6027 | α_2_ | 0.3973 |
|  | BET (m^2^/g) | 2016.87 | Pore Vol. (cm^3^/g) | 93 |
| MIL-101-Li  q*(cm^3^/cm^3^) = 650 | ε_o1_ (J/mol) | 6861.0 | ε_o2_ (J/mol) | 1450.6 |
|  | m1 (J/mol) | 2456.1 | m2 (J/mol) | 259.9 |
|  | α_1_ | 0.6492 | α_2_ | 0.3508 |
|  | BET (m^2^/g) | 2319.78 | Pore Vol. (cm^3^/g) | 1.16 |
| MIL-101  q*(cm^3^/cm^3^) = 1100 | ε_o1_ (J/mol) | 6866.9 | ε_o2_ (J/mol) | 1390.7 |
|  | m1 (J/mol) | 2441.5 | m2 (J/mol) | 255.1 |
|  | α_1_ | 0.6536 | α_2_ | 0.3464 |
|  | BET (m^2^/g) | 3402.69 | Pore Vol. (cm^3^/g) | 1.59 |

Table 2: Key values of parameters of SBA 15 - Nitrogen isotherm

| **Material Type** | **Parameter** | **Value** | **Parameter** | **Value** |
| --- | --- | --- | --- | --- |
| NNN/SBA  q*(mmol/g) = 9.46 | ε_o1_ (J/mol) | 1507.4 | ε_o2_ (J/mol) | 357.8 |
|  | m1 (J/mol) | 525.8 | m2 (J/mol) | 25.3 |
|  | α_1_ | 0.5343 | α_2_ | 0.4657 |
|  | BET (m^2^/g) | 306 | Pore Vol. (cm^3^/g) | 0.41 |
| NN/SBA  q*(mmol/g) = 12.6 | ε_o1_ (J/mol) | 1742.8 | ε_o2_ (J/mol) | 361.9 |
|  | m1 (J/mol) | 651.4 | m2 (J/mol) | 29.8 |
|  | α_1_ | 0.4813 | α_2_ | 0.5187 |
|  | BET (m^2^/g) | 354 | Pore Vol. (cm^3^/g) | 0.45 |
| N/SBA  q*(mmol/g) = 15.6 | ε_o1_ (J/mol) | 1679.8 | ε_o2_ (J/mol) | 368.3 |
|  | m1 (J/mol) | 715.8 | m2 (J/mol) | 19.8 |
|  | α_1_ | 0.5089 | α_2_ | 0.4911 |
|  | BET (m^2^/g) | 433 | Pore Vol. (cm^3^/g) | 0.55 |
| SBA-15  q*(mmol/g) = 19.8 | ε_o1_ (J/mol) | 2107.5 | ε_o2_ (J/mol) | 433.7 |
|  | m1 (J/mol) | 645.8 | m2 (J/mol) | 42.7 |
|  | α_1_ | 0.5183 | α_2_ | 0.4817 |
|  | BET (m^2^/g) | 670 | Pore Vol. (cm^3^/g) | 0.73 |

Table 3: Key values of parameters of MCM 41 N/Varients - Nitrogen isotherm

| **Material Type** | **Parameter** | **Value** | **Parameter** | **Value** |
| --- | --- | --- | --- | --- |
| NNN/MCM  q*(mmol/g) = 13.1 | ε_o1_ (J/mol) | 2889.3 | ε_o2_ (J/mol) | 827.3 |
|  | m1 (J/mol) | 974.7 | m2 (J/mol) | 67.7 |
|  | α_1_ | 0.5758 | α_2_ | 0.4242 |
|  | BET (m^2^/g) | 463 | Pore Vol. (cm^3^/g) | 0.24 |
| NN/MCM  q*(mmol/g) = 17.5 | ε_o1_ (J/mol) | 2854.7 | ε_o2_ (J/mol) | 820.2 |
|  | m1 (J/mol) | 1029.7 | m2 (J/mol) | 84.1 |
|  | α_1_ | 0.5769 | α_2_ | 0.4231 |
|  | BET (m^2^/g) | 686 | Pore Vol. (cm^3^/g) | 0.43 |
| N/MCM  q*(mmol/g) = 19.6 | ε_o1_ (J/mol) | 2937.7 | ε_o2_ (J/mol) | 830.0 |
|  | m1 (J/mol) | 1000.8 | m2 (J/mol) | 83.9 |
|  | α_1_ | 0.5675 | α_2_ | 0.4325 |
|  | BET (m^2^/g) | 701 | Pore Vol. (cm^3^/g) | 0.55 |
| MCM 41  q*(mmol/g) = 23.1 | ε_o1_ (J/mol) | 2865.0 | ε_o2_ (J/mol) | 832.6 |
|  | m1 (J/mol) | 973.1 | m2 (J/mol) | 85.9 |
|  | α_1_ | 0.5753 | α_2_ | 0.4247 |
|  | BET (m^2^/g) | 739 | Pore Vol. (cm^3^/g) | 0.58 |

Table 4: Key values of parameters of MCM 41 V/Varients - Nitrogen isotherm

| **Material Type** | **Parameter** | **Value** | **Parameter** | **Value** |
| --- | --- | --- | --- | --- |
| MCM-41-V3  q*(cm^3^/g) = 400 | ε_o1_ (J/mol) | 1255.1 | ε_o2_ (J/mol) | 843.3 |
|  | m1 (J/mol) | 1434.6 | m2 (J/mol) | 56.8 |
|  | α_1_ | 0.7402 | α_2_ | 0.2598 |
|  | BET (m^2^/g) | 938 | Pore Vol. (cm^3^/g) | 0.61 |
| MCM-41-V2  q*(cm^3^/g) = 270 | ε_o1_ (J/mol) | 3131.9 | ε_o2_ (J/mol) | 889.9 |
|  | m1 (J/mol) | 1662.2 | m2 (J/mol) | 128.3 |
|  | α_1_ | 0.5870 | α_2_ | 0.4130 |
|  | BET (m^2^/g) | 508 | Pore Vol. (cm^3^/g) | 0.43 |
| MCM-41-V1  q*(cm^3^/g) = 235 | ε_o1_ (J/mol) | 7137.4 | ε_o2_ (J/mol) | 982.2 |
|  | m1 (J/mol) | 5816.1 | m2 (J/mol) | 281.8 |
|  | α_1_ | 0.6227 | α_2_ | 0.3773 |
|  | BET (m^2^/g) | 475 | Pore Vol. (cm^3^/g) | 0.38 |

Table 5: Key values of parameters of MCM 41-PE Varient - Nitrogen isotherm

| **Material Type** | **Parameter** | **Value** | **Parameter** | **Value** |
| --- | --- | --- | --- | --- |
| MCM-41-PE  q*(cm^3^(STP)/g) = 1300 | ε_o1_ (J/mol) | 2001.2 | ε_o2_ (J/mol) | 200.9 |
|  | m1 (J/mol) | 718.1 | m2 (J/mol) | 37.9 |
|  | α_1_ | 0.2835 | α_2_ | 0.7165 |
|  | BET (m^2^/g) | 1230 | Pore Vol. (cm^3^/g) | 11.7 |
| MCM-41  q*(cm^3^(STP)/g) = 700 | ε_o1_ (J/mol) | 2589.1 | ε_o2_ (J/mol) | 701.8 |
|  | m1 (J/mol) | 1093.9 | m2 (J/mol) | 57.1 |
|  | α_1_ | 0.5104 | α_2_ | 0.4896 |
|  | BET (m^2^/g) | 1490 | Pore Vol. (cm^3^/g) | 3.3 |
